# Muscling in and Making Space: ‘Demonstrable Claims’ and ‘Jurisdictional Clipping’ in the Reconfiguration of Professional Jurisdictions in the Surgical Care of Older People

**DOI:** 10.1111/1467-9566.70003

**Published:** 2025-02-14

**Authors:** Justin J. Waring, Graham P. Martin

**Affiliations:** ^1^ School of Social Science and Humanities Loughborough University Loughborough UK; ^2^ The Healthcare Improvement Studies Institute University of Cambridge Cambridge UK

## Abstract

This paper examines the micro‐processes of jurisdictional change in the eco‐systems of healthcare work. This qualitative study investigated the expansion of geriatrician involvement in the perioperative pathway for older people. This study shows how, in response to opposition from surgeons and anaesthetists, geriatricians developed linked strategies that involved claiming the medical needs of surgical patients, and simultaneously integrating geriatric expertise into the non‐surgical peripheries of the pathway. By progressively demonstrating their ability to mitigate risks and improve surgical outcomes, geriatricians acquired an expanded role in the care pathway. This paper develops the concepts of ‘demonstrable claims’ and ‘jurisdictional clipping’ to explain the strategies of jurisdictional expansion. It also problematises these strategies by suggesting that role expansion was controlled and contained by more powerful incumbent groups, whereby the expansion of work was limited to temporal and spatial peripheries that were less valued by surgeons or anaesthetists.

## 1 Introduction

Faced with a healthcare workforce shortage (Buchan et al. 2023), policymakers are calling for innovations in healthcare work, including the deployment of new or extended roles, increased inter‐professional working, and use of digital technologies (McPake et al. 2024). Such change can disrupt the fragile eco‐system of healthcare work, as professions seek to create, defend and challenge jurisdiction over evolving healthcare tasks (Allen 2000; Bucher et al. 2016; Currie, Finn, and Martin 2009; Martin, Currie, and Finn 2009; Nancarrow and Borthwick 2005; Reay, Golden‐Biddle, and Germann 2006). Our study contributes to the existing literature on the changing eco‐systems of healthcare work by examining how professions *target* discursive claims at perceived deficiencies or ‘weak spots’ in existing jurisdictions, *and* simultaneously work to expand and integrate their expert practice at corresponding sites within the prevailing work system.

The study presented in this paper investigated attempts by geriatricians to expand their jurisdiction in the perioperative pathway for older people. Older people represent a growing proportion of surgical patients, where age‐related frailty, physiological deterioration and cognitive impairments can negatively impact surgical outcomes (Partridge et al. 2018). The integration of geriatric medicine into the perioperative pathway can offer multiple benefits for assessing health needs, optimising patients in advance of surgery, and managing recovery after surgery (Rogerson, Partridge, and Dhesi 2019). However, such role expansion disrupts the longstanding jurisdictions of surgeons and anaesthetists (Fox 1992). In response to initial opposition from surgeons and anaesthetists, the study shows how geriatricians expanded their role by making, what we term, ‘demonstrable claims’ about their ability to mitigate the medical risks of older patients, and by integrating their expertise into the peripheries of the surgical pathway as a form of ‘jurisdictional clipping’. Recognising the influence of prevailing professional hierarchies, we qualify these strategies by observing that claims to expertise were often based upon more determinate or technical expertise with a bounded contribution to improving surgical care, and that incumbent surgeons and anaesthetists were ready to delegate peripheral tasks because it released capacity to bolster their core specialist tasks and reinforce their superordinate status. An additional contribution of our study is its focus on professional eco‐systems characterised by mutually dependent or symbiotic jurisdictions. Although symbiotic jurisdictions have been described in the literature (Liu 2011), few studies have examined how they might become changed as part of workforce innovation.

### 1.1 Changing Systems of Expert Work

Abbott's (1988, 2005) eco‐systems perspective explains how expert occupations compete to create, challenge and defend jurisdiction over problems and tasks. Jurisdiction bestows control of clients, authority within the division of labour and professional status. His work invokes ecological metaphors to describe professions as ‘*carnivorous competitors that grow in strength as they engulf jurisdictions*’ (Abbott 1988: 87). Abbott describes how claims to jurisdiction are made with various public, legal and professional audiences, typically based upon an occupation's specialised knowledge that enables it to ‘*classify a problem*, *reason about it*, *and take action on it*’ (Abbott 1988: 40). The outcomes of competition vary from full closure or exclusive control to negotiated settlements where one profession is subordinate to another, or where there is a division of labour based on client segmentation and functional separation (Timmermans 2002). Through jurisdictional settlement, patterns of professional hierarchy and inter‐dependency are created as an eco‐system of expert work, reflecting structural disparities in status and power (Abbott 1988).

Although Abbott's theory foregrounds occupational competition, it also recognises the inter‐dependencies common to many expert systems. Of relevance to our research are the micro‐processes through which collaborative jurisdictional settlements are forged. Here, we invoke another ecological concept—‘symbiosis’—to consider how some eco‐systems are characterised by inter‐professional dependence (Liu 2011). Here, we elaborate this concept to describe situations where, for example, a task cannot be completed by one expert alone and so requires the direct involvement of two (or more) occupations with different forms of expertise, or situations where one expert relies on the indirect involvement on another to fulfil their task. More than collaboration or co‐working, the key distinguishing feature of symbiosis is that each occupation depends upon the other for the maintenance of its own jurisdiction. The concept of symbiosis is exemplified by the inter‐dependencies between surgeons and anaesthetists in the organisation of surgery (Fox 1992). How such relationships emerge, evolve or dissolve over time is little understood in the existing literature on professions.

An extensive literature examines how professionals compete for jurisdiction and ‘work out’ collaborative settlements (Adler, Kwon, and Heckscher 2007; Allen 2000; Blok et al. 2019; Kellogg 2014; Langley et al. 2019; Reay, Golden‐Biddle, and Germann 2006). Following Abbott (1988), many studies take a primarily discursive orientation looking at the claims made with different audiences. Sanders and Harrison (2008), for example, analyse the ‘legitimation talk’ of cardiologist, geriatricians, nurses and general practitioners in justifying their distinct expertise in heart failure services. Bucher et al. (2016) similarly describe how professional associations use framing strategies that construct task jurisdictions to align with their expertise and identity. Such discursive claims typically rest upon a profession's claims to expertise that meet the expected standards or assurances of different audiences. Although Abbott (1988) emphasised the importance of abstract knowledge, more recent research suggests other forms of evidence can underpin jurisdictional claims. For example, Baeza et al. ’s (2016) study on stroke reconfiguration found that subordinate occupations benefit from advances in evidence‐based practice, but that more dominant groups continue to determine the standards of evidence to cement their position. However, it might be argued that much of this literature tends to analyse the role of discursive claims in isolation, such as textual or verbal proclamations, without also looking at how such claims interact with other discourses, such as counterclaims, or function as part of broader interactive activities. Furthermore, there is little attention to how claims are targeted at perceived deficiencies in the day‐to‐day allocation of tasks and roles.

Looking closer at shopfloor interactions (the level of profession*als* rather than professions), a parallel literature examines how jurisdictions are routinely negotiated by, for example, finding trading zones, negotiating routines and bridging boundaries between occupations (Allen 2008; Blok et al. 2019; Currie, Finn, and Martin 2009; Liberati, Gorli, and Scaratti 2016; Kellogg 2014; McDonald et al. 2010; Oh 2014; Powell and Davies 2012). This research suggests that jurisdictional disputes are inherently situated, interactive and focused on specific task domains. Reay, Golden‐Biddle and Germann (2006) describe the micro‐processes of jurisdictional change as a series of incremental activities aimed at cultivating new opportunities, achieving quick wins and demonstrating value. Elaborating this idea, it seems rare that a profession will seek to challenge the entire jurisdiction of an incumbent profession; rather, such challenge tends to be targeted at specific tasks, clients or settings. In thinking about how jurisdictional challenges might be targeted, we observe that jurisdictional settlements are often demarcated by time and place (Abbott 1988). For example, Allen (2002) shows how the social organisation of daily tasks amongst nurses and other healthcare professions is structured according to the spatial and temporal organisation of care work. It might be expected, therefore that jurisdictional disputes will have a temporal‐spatial dimensions related to not only ‘who’ undertakes tasks but ‘where’ and ‘when’. To date, this aspect of jurisdictional change remains relatively under‐researched.

Our study contributes to this literature by asking how jurisdictional change is realised through professional groups targeting specific deficiencies or ‘weak spots’ in incumbent jurisdictions, manifest through both making discursive claims and expanding their expert practices into specific times and places of the work eco‐system. We recognise, however, that jurisdictional disputes are shaped by prevailing social hierarchies within the work eco‐system (Oh 2014). For example, Martin, Currie and Finn (2009) show how the expansion of extended roles amongst general practitioners was shaped by hospital specialist who used their superordinate position to limit role expansion to more peripheral tasks. Therefore, it is important to analyse jurisdictional disputes in terms of actors' unequal social positions within prevailing hierarchies, recognising that these will shape the outcome of efforts to make new jurisdictional claims and expand work practices.

### 1.2 The Surgical Care of Older People

An aspect of healthcare work that exemplifies professional symbiosis is surgery (Fox 1992). Anaesthetists and surgeons bring distinct expertise to the work system, but each depends (but not entirely) on the other to carry out its work and maintain its jurisdiction. The surgeon cannot operate without the anaesthetist providing pain relief, inducing sleep, numbing sensation and monitoring patient health. Without invasive surgical care, there would be less demand for anaesthetic expertise (acknowledging that anaesthetists have expanded their professional project beyond surgery—Green et al. 2011). The last decade has seen calls for increased multi‐professional working across the surgical pathway, especially in the surgical care of older people (Centre for Perioperative Care 2021). In addition to surgical pathology, older people often have age‐related co‐morbidities, frailty and cognitive decline, which are associated with postoperative complications (Partridge et al. 2018; Rogerson, Partridge, and Dhesi 2019). Traditionally, such issues are assessed in the preoperative period by the surgical and anaesthetic teams and, where necessary, through referral to a geriatrician. Over the last decade, the field of perioperative medicine has advocated for a more integrated role for geriatricians (Centre for Perioperative Care 2021; Dhesi, Moonesinghe, and Partridge 2019; Partridge et al. 2018). A prominent example is the involvement of ortho‐geriatricians in older people's orthopaedic surgical pathway (Aw and Sahota 2014). Other models of ‘geriatric liaison’ have emerged in countries, including Australia, Canada, the US and the UK (Mistry et al. 2023; Partridge et al. 2014). Geriatric involvement can encompass the entire surgical pathway often using the comprehensive geriatric assessment (CGA) to provide a multidimensional holistic assessment of an older person's medical, physiological, functional and social circumstances (Dhesi, Moonesinghe, and Partridge 2019; Saripella et al. 2021). Preoperatively, the patient is assessed to support shared decision‐making about the relative benefits and risks of surgery, including the option to decline surgery, or to optimise fitness for surgery and recovery through recommending interventions, for example, smoking cessation and medicines optimisation. Postoperatively, geriatricians collaborate with the surgical team to formulate plans for rehabilitation and discharge thereby reducing the risk of unplanned readmission and enhancing recovery. The development of such services reflects the longer history of geriatric medicine, whose position in healthcare has waxed and waned in the context of wider social and political changes (Pickard 2010), but whose position now seems integral to meeting the needs of an ageing population and addressing wider developments in professional discourse, such as patient‐centeredness and shared decision‐making.

The expanded role of geriatricians in the perioperative pathway has the potential to disrupt the eco‐system of professional work, especially the symbiotic relationship between surgeon and anaesthetists. Returning to the debates outlined above, there is a need to understand geriatricians' work to expand their role in the perioperative pathway, especially how they target their claims to expertise and integrate their practices with specific task domains.

### 1.3 The Study

The paper reports on a qualitative study carried out between 2022 and 2023 that investigated the introduction of geriatrician services in perioperative pathways for older people. The study received favourable ethical review by the University of Birmingham Research Governance Service in September 2022.

We first carried out a national‐level interview study to investigate the social history of perioperative services for older people. Interviews were carried out with 33 people, including leaders in geriatric medicine (11), surgery (8) and anaesthetics (5), and representatives from general practice (2), nursing (2), organisational development (3) and patient representative groups (2). All participants were contacted via email or by referral. The topic guide covered participants' views about the development of geriatric services in the surgical pathway, changing professional relationships, and the evolving evidence‐base for such services. This was complemented by a documentary analysis of the research and policy literature on perioperative services for older people. This included over 30 academic papers and policy documents that were reviewed narratively, and a desk review of NHS services' website and online policies to understand how services were configured locally.

We next investigated efforts to integrate geriatrician care into the perioperative pathways of older people in nine different NHS hospitals. At the hospital level, these services tend to be led by a small number of geriatricians (2–4 per site), and so sampling aimed to gather comparable insight from multiple hospitals to understand common and diverse experiences. Following the above desk review, seven hospitals were initially recruited considering factors such as size, research/teaching profile, geographical location, number of surgical pathways, and length of time geriatric services had been integrated into the perioperative pathway. Following feedback from study advisors, two additional sites were recruited that had been unsuccessful in implementing a geriatric perioperative pathway, to provide further comparison (see Table 1). At each site, initial contact was made with the lead geriatrician, who was asked to identify representatives from surgery, anaesthesia, nursing and management. An important limitation, therefore, is that the selection of participants was potentially biased by the preferences and connections of the lead geriatrician, which might also account for the uneven representation of surgeons across case study sites. As shown in Table 2, there were variations in the range of professional representatives across study sites, which also reflected differences in how services were configured locally, especially whether they were geriatrician, anaesthetist or nurse‐led services. In total 36 people participated in interview during this stage of the study.

**TABLE 1:** Study site characteristics.

Site	Setting	Type	Profile	Length of time
1	Metropolitan > 2 million pop.	Teaching/Specialist	> 1500 beds > 23,000 staff	Over 5 years
2	Metropolitan > 1 million pop.	Teaching/Specialist	> 600 beds > 13,000 staff	Over 5 years
3	City > 600,000 pop.	District general hospital	> 900 beds > 6500 staff	2–5 years
4	City > 450,000 pop	Teaching/Specialist	> 1000 beds > 14,000 staff	2–5 years
5	City > 400,000 pop.	District general hospital	> 700 beds > 5000 staff	Less than 2 years
6	City > 500,000 pop	District general hospital	> 450 beds > 3000 staff	Less than 2 years
7	Metropolitan > 700,000 pop	Teaching specialist	> 700 beds > 9000 staff	Less than 2 years
8	City > 150,000	District general hospital	> 500 beds > 4000 staff	Failed adopter
9	City > 290,000	District general hospital	> 750 > 7000 staff	Failed adopter

**TABLE 2:** Study participants at hospital sites.

Site	Geri.	Surgeons	Aneas.	Nurses	Mgmt.	Total
1	3	3	1			7
2	3	1		1		5
3	3					3
4	1	1	3		1	6
5	1			2		3
6	1		1		1	3
7	1	1	2			4
8^a^	2				1	3
9^a^	1		1			2
Total	16	6	8	3	3	36

^a^ Sites unsuccessful in introducing perioperative geriatric service.

Data collection involved semi‐structured interviews, using topic guides covering the rationale for developing perioperative geriatric services, the process of introducing such services and attitudes towards changing roles and responsibilities. Interviews last between 40 and 120 min and were audio‐recorded with consent and transcribed. Additionally, observations were carried out of team meetings, training events, and national professional events across four sites. This included around 40 h of observations of a dedicated training programme for perioperative geriatric services and two national conferences and special interest group meetings. These observations afforded insight into the way new roles were explained with different public and professional audiences. Observation notes were recorded and integrated into the larger data set.

**FIGURE 1:**
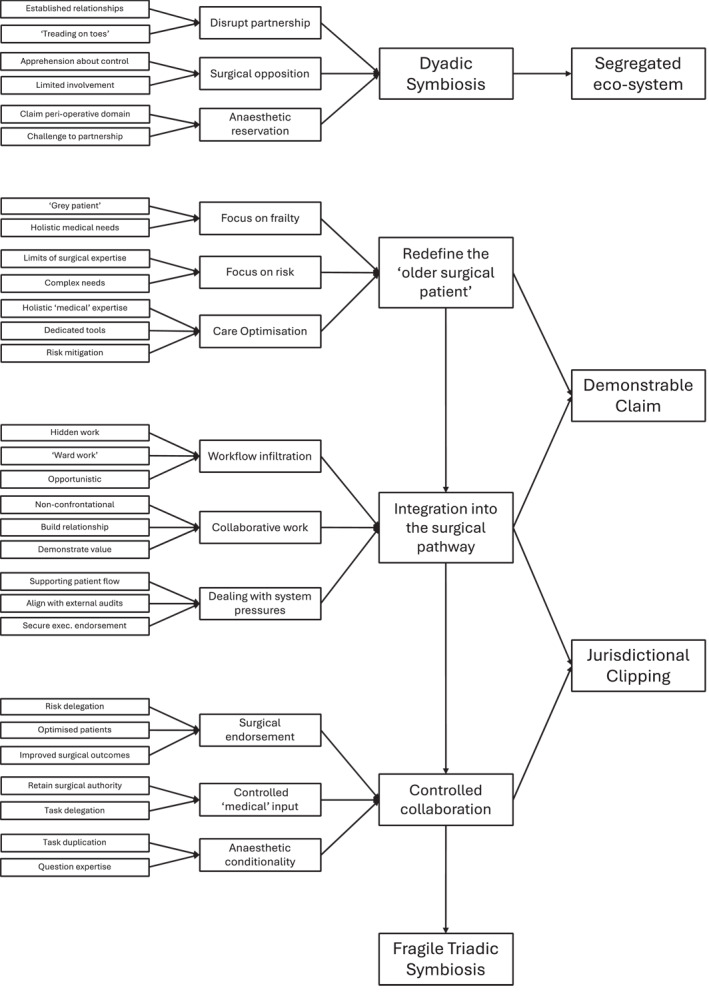
Visualisation of coding and thematic analysis.

Data analysis followed an inductive method of incremental interpretation and thematic analysis (Corbin and Strauss 1990; Gioia, Corley, and Hamilton 2013), followed by re‐engagement with the wider literature. Data were initially subject to open reading to code sections of data relevant to the research focus. These preliminary codes were sifted and sorted following the principles of constant comparison with a focus on internal cohesion, conceptual connections and analytical relevance. These initial steps were carried out by JW with GM reading a sample of transcripts and reviewing codes for consistency. The first‐order codes were then re‐analysed by both authors following the principles of axial coding to examine similarities and differences and to produce second‐ and third‐order codes. Through this process, data were themed according to how geriatricians expanded their jurisdiction in the perioperative pathway by target their jurisdictional claims and integrating their practice into the care processes in response to surgeons' and anaesthetists' initial opposition. These themes were related back to the existing literature on the social organisation of healthcare work to sharpen and refine interpretation (Figure 1).

### 1.4 Findings

Many geriatricians talked about their speciality's quest to establish a valued position in the healthcare division of labour (see Pickard 2010). Contemporary concern with an ageing population was commonly used to justify role expansion, but existing structures of care were often viewed as thwarting desired change. Talking at a national event, one senior leader talked of geriatricians needing ‘*slightly sharper elbows to influence decision makers and improve health and care for older people*’. This idea of ‘muscling in’ was a prominent theme in this study but so too was the need to deal with the responses of surgeons and anaesthetists on the shopfloor.

#### 1.4.1 Disrupting the Perioperative Pathway

Geriatricians' strategies for expanding their jurisdiction in the perioperative pathway need to be understood in the context of the wider structures of professional power and status, as manifest in the initial opposition of surgeons and anaesthetists. Representatives of all three professional groups acknowledge the expansion of geriatrician involvement across the perioperative pathway disrupted the prevailing model of care. As one geriatrician said: ‘*you're treading on other people's toes all the time*’ [1.2Geriatrician [G]]:

This is disruption to usual practice. You've had surgical and medical care very separated out in hospitals, and anaesthesia viewed very much as a service specialty for surgery … people are very used to sitting within the structures and their teams, and can feel threatened when you're suggesting a change to that approach. … And so, people do feel sometimes like, you know, treading into my territory. [1.1G]

Surgeons' initial reactions to increased geriatrician involvement seemed to be grounded in a sense of anxiety about the challenge to surgical decision‐making and a corresponding loss of autonomy:

I suppose the fear is, [surgeons] think that [geriatricians] would start interfering with the surgical management of the patients… It's not for them to manage the surgery. [1.14 Surgeon[S]]

I’ve always thought of myself as a clinician who takes a lot of ownership over their patient, and I’ve always wanted to be totally in charge with regard to surgical management of patient …. Frankly, when [named geriatrician] first came I was a bit, not resentful, but very cautious of another clinician making decisions on patients without my direct involvement… Another clinician not directly part of my chain of command coming in, making decisions, altering medicine etc, I was a little bit sceptical in terms of the ownership of the patient. [3.4.4S]

Senior leaders within anaesthesia commented that the field of perioperative care (with its focus on the whole pathway) was broader than geriatric medicine and was born of anaesthetic practice. In this way, they appeared to stake a jurisdictional claim over this emerging field of practice.

[We] developed peri‐operative programmes out of anaesthetics…. A lot of us thought that to serve up the right patient at the right places at the right time for the right operations, you've got to have the conversations beforehand. So that's really how the perioperative programme started to develop. [1.25 Anaesthetist[A]]

Across all sites, such opposition was manifest in geriatricians being excluded by surgeons and anaesthetists in key decision‐making stages, such as assessment clinics or post‐surgical ward rounds (despite efforts from other professional groups to engage geriatricians). Surgeons more often preferred geriatrician involvement to be based on referral and consultation, rather than shared decision‐making. This enabled surgeons to contain and control the involvement of geriatricians around complex cases. As found in the two ‘unsuccessful’ hospital sites, geriatrician involvement continued to be based on referral for advice or following formal handover of care to support discharge. Such ways of working reflected a more demarcated division of labour, in which surgeons retain primary decision‐making authority.

The initial expansion of geriatricians across the perioperative pathway was therefore met with opposition from the two incumbent professions, exemplifying what Abbott (1988) would see as a competitive territorial dispute. The defensive boundary work of surgeons and anaesthetists resembled a kind of alliance, with surgeons using their overarching structural authority to limit geriatrician involvement, whereas anaesthetists claimed relevant expertise to competently perform many aspects of non‐surgical perioperative care. This type of defensive boundary work is well‐documented (Langley et al. 2019), but our study shows how surgeons and anaesthetists defended their respective jurisdictions in a coordinated way thanks to their symbiotic bond.

In response to such opposition, geriatricians developed targeted strategies to better integrate their expertise in the perioperative pathway. In the longest functioning study sites, these appear to have emerged organically, but thanks to the sharing of experience at professional networks, geriatricians in other study sites seemed to be taking a more planned and standardised approach. However, such strategies need to be understood as occurring against a backdrop of continued surgical dominance across the perioperative pathway and, significantly, medico‐legal accountability for the care of the patient.

#### 1.4.2 Claiming the Older Surgical Patient

The first strategy deployed by geriatricians involved redefining and claiming the underlying health problems presented by the older surgical patient. Accepting that surgical patients typically had disease requiring surgical intervention, geriatricians called for a more holistic view of the health needs associated with age‐related frailty. In line with Abbott (1988), geriatricians used their distinct expertise to re‐classify, reason about and act upon the older surgical patient. Several participants talked of the ‘*grey patient*’, with two complementary meanings for establishing their expertise. The phrase distinguished older patients based upon their age and frailty, but also the multi‐dimensional ambiguity and complexity of their care needs—and hence the relevance of geriatric expertise to determine and address those needs:

Some of these patients are quite grey… I think when you have that realisation that lots of patients don't sit in a box… The grey patient. They just don't sit neatly anywhere. [1.6G]

Geriatricians elaborated this complexity through the discourse of risk, in which surgeons' lack of ‘medical’ expertise relevant to an older person's needs could jeopardise the success of surgery and, by implication, the standing of the surgeon.

If your job is to fix the bladder, or to fix the bone, or to fix whatever, some people find it difficult to put that in the context of everything else…. The problem is a huge number of complicated patients, some of whom need surgery, some of whom don’t, who could probably do better if we sorted out their medical problems. [1.2G]

They are basically frail people … with a whole load of medical problems which were actually very familiar to us, but very unfamiliar to surgeons. [1.6G]

Geriatricians then positioned themselves as able to mitigate such risks and thus alleviate the anxiety that surgeons might face in decision‐making. They advanced their input through their use of diagnostic tools, such as the CGA, and the application of evidence‐based interventions to optimise health. Such tools and resources facilitated geriatricians' claims to ‘optimise’ older patient ahead of surgery and mitigate risk, thereby contributing to the success of surgery and enhancing the standing of surgeons.

[The] geriatrician‐led approach, which is tailored to the complexity and multimorbidity of both the patient and to the pathway … helps navigate that pathway for both other clinical stakeholders and for the patient stakeholders, in order to try and effect the best outcome. [1.2G]

Your senior surgeon will make you a good surgeon, but I [as a geriatrician] will make you an excellent surgeon, because I'll make you think more about your patient. If your patient is not eating or drinking, the wound is not healing…and that's the mentality that we're trying to teach these in individuals to try and manage people a little bit more holistically. [1.23G]

Geriatricians made these claims in various settings with surgical, anaesthetic and managerial audiences, including clinical audit activities and departmental team meetings. In concert with the activities described below, geriatricians made purposeful efforts to use routine service data and surgical outcome data, such as length of stay and 30‐day mortality rates, to demonstrate the value of their involvement. This also included claims to enhance patient satisfaction, with geriatricians asserting a closer and more holistic understanding of patient preferences, thereby allowing them to involve patients in more meaningful shared decision‐making.

‘There's been more of a focus on the need for making, helping, working with patients to make the right decisions for them, and so more of a focus on shared decision making, … So that was something that we felt we are doing’ [1.1G]

I think it’s been such a niche that we’ve been missing that’s now there…that communication and presence…the joint surgical and medical team. [2.5G]

Although surgeons initially opposed such influence in surgical decision‐making, it seemed that many came to regard geriatricians as a mediator between the surgical team and the patient. Many surgeons reflected how, over time, and with growing interaction with geriatricians, they had come to acknowledge the complexity of frail older patients and thus accept increased geriatrician involvement for mitigating risk and improving surgical outcomes.

Surgical culture is now [about] how many do you do, what is your mortality rate. Your 30 days….and then you can see that speed is important because that way you get to do more and having someone else to offload the risk and mitigate the risk. [1.9S]

Over time, and in concert with the practices described below, the foundations of a mutually beneficial arrangement were forged in which geriatricians' involvement became valued by surgeons for dealing with medical care needs at the edge or beyond the scope of their expertise. However, there was an important demarcation between medical and surgical expertise, as distinct specialities. Specifically, geriatricians' expertise was commonly viewed as more general and determinate in character, and based on the application of protocolised evidence‐based procedures, thereby making it seem less specialised than surgical expertise. Geriatrician involvement might be viewed as a way to delegate the risk of ‘medical’ complications, but many surgeons continue to believe that such expertise needed to be deployed under surgical supervision. Hence, geriatricians' expanded role appeared to be sanctioned and controlled by surgeons as the dominant incumbent group.

It's been a very long time since I've done medicine and, you know, things have moved on quite a bit, from when I was at medical school… we're not medics. This is this is the stuff which is outside of our expertise… there are definitely people who fall in the grey area. [1.14S]

There are lots of sources of information that [geriatricians] have to interpret and make sense of to give you a steer or idea of what will happen next…and as a surgeon who doesn’t really know the patient, you can just look at that [information] and you can say ‘fly or no fly’. [1.9S]

Thus for some surgeons, geriatricians were adopting a ‘handmaiden’ role—but one which, through the incremental servicing of surgical patients' medical needs, lay the foundations for dependency.

#### 1.4.3 Infiltrating the Perioperative Pathway

In concert with above, geriatricians engaged in practical activities to demonstrate their value to and gain traction within the perioperative pathway. Recognising the structural authority of surgeons and with firsthand experience of their ability to oppose and block involvement, geriatrician leaders described playing a ‘long game’ of cultivating inter‐personal relationship, continually demonstrating the value of their involvement, building trust and, importantly, avoiding confrontation:

You have to play a long game … as a cuckoo in a nest … because you just ruffle everyone's feathers, and everyone will hate you. [1.2G]

Across many of the study sites, geriatricians described a form of jurisdictional infiltration in which they inveigled themselves into under‐served or uncontested areas of the pathway. In several sites, this involved working with nursing staff in outpatient clinics to complete the preliminary review of patient history, to assess frailty and cognitive impairments, and offer advice on risks to surgery. In other sites, geriatricians visited surgical wards at times when there was limited senior surgical presence to support surgical trainees by reviewing patient records, completing medicine reviews, and supporting discharge planning. These early stages of integration seemed more covert or ‘under the radar’ (Reay, Golden‐Biddle, and Germann. 2006) and concerned with avoiding direct confrontation. Furthermore, such activities were often aligned with the work of other or less experienced professional groups as a way of providing medical input, but without challenging the incumbent specialists. Over time, however, both anaesthetists and surgeons began to recognise and value such involvement.

And what we’re in the business of, and this is in a sense a Trojan horse…it’s been opportunistic and piecemeal and iterative. [1.6G]

We'd been on the wards for a while, and there was a couple of consultants that we got on really well with, but generally I would just get my head down and go and see the patients, and work hard clinically, and not talk too much, and I didn't even know some of the surgeons. [2.1.1G]

As the benefits of geriatricians' involvement became recognised, their activities became more overt and oriented towards formal integration into the perioperative pathway. This involved, for example, being invited to join surgical decision‐making meetings and attending ward rounds. In some sites, it also involved coordinating surgical outpatient clinics with geriatric assessment clinics, and in one site having a combined preoperative assessment clinic.

I think, as we've done more and been more visible on the wards, people have understood more what it can add, and allows the team to grow. …You know that presence and ambassadorial feature. So, when I walk onto surgical wards, you see all the heads turn. I think it’s about owning the environment. [3.1.1G]

Through these tactics, geriatricians could directly demonstrate their ability to improve surgical outcomes and patient recovery:

In a kind of back door where you'd slightly embedded yourself …. it was a sort of organic thing, but based on a lot of presenting data and a lot of just getting to know people and constantly having the same conversations. [1.2G]

Geriatricians also aligned their efforts with broader policy developments and national audits. In particular, the National Emergency Laparotomy Audit (NELA) showed that surgical services had better outcomes when a geriatrician was integrated across the perioperative pathway, which informed national recommendations. This placed additional external expectation on surgeons to recognise the value of geriatricians:

We stuck to NELA patients initially, and no one could argue that we were coming along to see their patients, because it was recommended that we would. [3.3.1G]

At the hospital level, geriatrician leaders also garnered endorsement of hospital managers by demonstrating their contribution to organisational performance:

When we're talking to directors or managers…we're going to emphasise how we improve the flow of the patient through the hospital currently because of other pressures…. improve, increase, reduce the time it takes for a patient to be more medically stable ready for discharge. [2.3.1G]

Taken together, geriatricians' efforts to integrate their expertise into the perioperative pathway seemed to involve a degree of subterfuge and infiltration at the peripheries of surgical and anaesthetic practice. This revealed an important temporal‐spatial aspects of jurisdictional expansion over tasks found at specific times and places, especially where incumbent professions were less active and present. Because surgeons regarded ‘ward work’ as peripheral and low status, they were amenable to ceding responsibility, but as we show below, they continued to retain overall control.

#### 1.4.4 Controlling the Expansion of Geriatric Involvement

Geriatricians talked about ‘winning over’ surgeons and, to a lesser extent, anaesthetists, as their role became increasingly valued:

Our most strong enemies in the first year are now our strongest allies. I’ll never forget. A surgeon came up to me and said, ‘You’re not telling me who I can fucking operating on’. And you know what, a few years later they’re banging on the door asking for help with that. [3.1.1G]

Many surgeons and anaesthetists reflected on how their views about geriatric involvement had become more positive:

When [named geriatrician] has come in, I have found it to be an absolutely brilliant both for me and my patients…I have certainly got a lot more through surgery with good quality lives afterwards. [3.4.4S]

We do need a geriatrician to support this service, and they need to be the ones that run this service, and we will then support decision‐making and support in the peri‐operative management. [3.6.3A]

However, such acceptance was conditional on where and when geriatrician input was deemed appropriate. In the following excerpts, for example, two surgeons continue to describe their relationship with geriatricians less in terms of equal partnership and more in terms of referral service in which the surgeon retained authority. Such language suggests a continuing hierarchy of authority in which geriatricians were subordinate, and in which surgeons continued to determine the boundaries of both professions' jurisdictions:

The surgical **liaison** service is excellent, they really help with the more complex older people. [1.26S]

I'm not saying I'm giving the responsibility to them to decide what to do on that patient. But I found this sort of integrated approach okay. [1.15S]

Ongoing surgical dominance was reinforced by legally‐underwritten structures of accountability that both enshrined both their position and their ability to delegate work to others. One surgeon neatly summarised this authority with reference to their ‘name’ being above the patient's bed:

I respect [their] autonomy and expertise, but my name is above the patient's bed, because I've done the operation … and she takes care of the bits that I can't take care of… they just come in and sort it all out for us. [1.14S]

Anaesthetists also grew to support geriatrician involvement, especially where it helped mitigate risks in the preoperative period. In some situations, it seemed that anaesthetists and geriatricians could collaborate to wield stronger influence over surgical decision‐making than when working separately, especially about whether a patient should proceed to surgery given their complex risks. Thus, in some circumstances, anaesthetists and geriatricians could form new alliances to counter the dominance of surgeons. However, many anaesthetists continued to harbour reservations about geriatricians' encroachment on their role in the surgical pathway. As with surgeons, there was a strong sense that geriatricians' involvement should be limited to pre‐determined complex patients and at certain points in the pathway—with the concomitant view that anaesthetists should retain control over the inter‐speciality relationship and delegate specific tasks, selected by them, to geriatricians.

I send them to our high‐risk peri‐operative clinic, and they will get seen by one of my geriatric colleagues…our plan would be to have a joint anaesthetic and geriatric clinic for these patients…So the referral is from the anaesthetic team, but most of the work is actually done by the geriatric team. [3.4.1A]

The temporal‐spatial conditionality of geriatricians' jurisdiction was further described by anaesthetists who argued that involvement should be limited to certain periods of the pathway to avoid duplication of the anaesthetist's role:

When [patients are] in the ICU, there's nothing that [geriatricians] could add, but once they discharge they go to the ward, then they could probably add a little, add more and have a look through the notes and see the patients and stuff like that. [3.9.1A]

Echoing the views of some surgeons, some anaesthetists questioned whether geriatricians' practice relied less on specialist expertise and more on the competence to apply particular tools, using skills that could just as easily be acquired by other professional groups:

You could take the core elements which would be the CGA, the MDT, the holistic, proactive, interdisciplinary care planning, but whether that's delivered by a geriatrician implanting themselves in the surgical pathway, or whether that's by existing members of the surgical team broadly configured. [1.8A]

Is this the right approach for every patient to have CGA? My view on that is probably not. I think you can screen patients effectively and there are an increasing number of tools that you can use to do that….the deep diver might be the CGA but it might be something else. [1.13A]

As one geriatrician reflected, the relationship with anaesthetists remained challenging, possibly because, at times, the boundary between their jurisdictions was unclear and with greater scope for competition. As with surgeons, geriatricians' incremental efforts to engage anaesthetists, especially by demonstrating their value to the overall pathway, enabled something of a truce.

The relationships with the anaesthesia have been challenging and probably still remain challenging… because there's also been a move at the same time within anaesthesia to working in perioperative medicine and viewing themselves as the perioperative physician. [WP1.1G]

Although surgeons and, to some extent, anaesthetists came to welcome the integration of geriatricians, this was conditional on retaining authority over the perioperative pathway and restricting geriatrician involvement to certain tasks found at certain times and places within the pathway. Therefore, the ceding of responsibility resembled a form of delegation and substitution (Nancarrow and Borthwick 2005), in that surgeons and anaesthetists used their superordinate positions to limit geriatricians' expansion in ways that not only mitigated risk but also benefited each to concentrate on, what they saw as, the core aspects of their roles.

## 2 Discussion

Our study examined how efforts to integrate geriatric expertise into the perioperative pathway for older people impacted on the eco‐system of professional work, especially the symbiotic relationship of surgeons and anaesthetists (Fox 1992). Focussing on the discursive and micro‐interactive processes of change, our paper explores how professional eco‐systems evolve over time, from an initial competitive phase of ‘muscling‐in’ in which geriatricians sought to expand their jurisdiction (albeit often surreptitiously), to a subsequent phase of ‘making space’ in which surgeons and anaesthetists ceded aspects of their jurisdiction to allow for more collaborative working whilst retaining overall authority.

Our study contributes to the existing literature by examining how jurisdictional change is manifest through the linked strategies of targeting discursive claims at perceived deficiencies in incumbent jurisdictions whilst simultaneously integrating practices at specific task domains. Although research shows how both discursive claims and shopfloor interactions contribute to creating, challenging and defending jurisdictions (e.g. Allen 2008; Liberati, Gorli, and Scaratti 2016; Kellogg 2014; Oh 2014), our work shows how this combined strategy enabled geriatricians to make ‘demonstrable claims’ that provided more substantive foundations for jurisdiction expansion. This was manifest through the ability to articulate *and* mitigate the risks of older surgical patients whose frailty could jeopardise surgical outcomes and, in turn, the standing of the surgeon. Although studies detail the construction and framing of discursive claims (e.g. Bucher et al. 2016), our concept of ‘demonstrable claims’ better accounts for how claims become mobilised in material practice.

Looking closer at the interactive practices of geriatricians, we delineate three strategies that involved (i) avoiding direct confrontation, (ii) working covertly at the peripheries of existing jurisdictions and (iii) demonstrating the value of collaboration. These broadly align with Reay, Golden‐Biddle and Germann (2006), especially the idea of working ‘under the radar’ and making ‘quick wins’. We add to this work by proposing the concept of ‘jurisdictional clipping’ to explain how jurisdictions are gained/eroded through incursion at the margins of territorial practice. We base this idea on the practice of ‘coin clipping’, where the peripheral margins of an entity are appropriated, in a barely perceptible way, leading to its devaluation. This can be seen with geriatricians' efforts to covertly and incrementally claim aspects of surgical (and to some extent anaesthetic) practice that were not central to either group, at least as understood by each profession. This jurisdictional clipping is seemingly enabled by the ‘taskification’ of expert work, that is, the disaggregation of complex activities into discrete functions that can be reallocated across occupational groups (Arcidiacono, Pais, and Piccitto 2023).

Although jurisdictional expansion can occur through the linked processes of making demonstrable claims and jurisdictional clipping, our analysis also leads us to problematise these strategies. This is grounded in the recognition that structural disparities in professional status continue to shape, but not determine, how jurisdictional disputes are manifest and resolved (Martin, Currie, and Finn 2009; Oh 2014). We first re‐consider the basis of geriatricians' demonstrable claims. These were largely based on their holistic understanding of the medical needs of older surgical patients, and expressed through the application of tools, such as the CGA. Surgeons and anaesthetists viewed this expertise as important, but also more technical and reducible, than their own specialised expertise. The apparent status hierarchies in expertise and their role in justifying professional jurisdiction, reflect what Jamous and Peloille (1970) and later Abbott (1988) discuss about the special significance of specialist, indeterminate or abstract knowledge in jurisdictional claims, relative to generalist, determinate or technical knowledge. Kessler, Heron and Dopson (2015) describe similar processes of specialist‐delegation and generalist‐hoarding, which reflect different logics around regulation and inter‐professional working. This echoes Baeza et al. ’s (2016) work on stroke reconfiguration in which nurses' use of evidence‐based practices facilitated role expansion, but did not reduce their subordinacy relative to doctors.

Turning to the concept of jurisdictional clipping, it might be assumed that appropriating the tasks held by another would devalue the jurisdiction of the incumbent to the benefit of the challenger. However, our study found that surgeons often regarded these ‘medical’ tasks as peripheral and less important than their core ‘surgical’ work, and were therefore willing to cede these tasks to geriatricians whilst maintaining control over the reallocation of work. Taking on ‘scut work’ has been shown to provide the foundations for enhanced occupational status (Huising 2015), but in this case any enhanced status for geriatricians was conditional on it being of advantage to surgeons, and to a lesser extent anaesthetists. Specifically, surgeons endorsed geriatrician involvement as it involved the delegation of ‘medical’ tasks, mitigated risk and enhanced surgical outcomes, whilst also freeing surgeons from unwanted tasks to concentrate on their core specialist tasks. Therefore, geriatricians' expansion was to the advantage of surgical practice and maintained the relative position of this incumbent profession (see also Currie, Finn, and Martin 2009; Nancarrow and Borthwick 2005). The clipping of anaesthetic practice was more disputed because anaesthetists saw many of these medical tasks, especially in the pre‐operative phase as much more central to their work. Future research might look further at jurisdictional clipping in other eco‐systems and its relationship with the increased taskification of professional practice.

Notwithstanding these points, across many case study sites the eco‐system of perioperative care for older people transitioned from a model defined by the dyadic symbiosis of surgeons and anaesthetists to an emerging model of triadic symbiosis with geriatricians integrated into perioperative pathway. However, this new triadic relationship was arguably conditional and partial, for at least three reasons. First, there were fewer directly shared or overlapping jurisdictions at the level of time and space, such as the shared space of the operating room for surgeons and anaesthetists. Second, the relationship was limited to only certain patients, that is, frail older people and not the entirety of the surgical client base. Third, the pattern of inter‐dependence resembled a more segmented or controlled division of labour that maintained the status of surgeons (Abbott 1988). More significantly, although the jurisdiction of surgeon and anaesthetists may have been enhanced with the inclusion of geriatricians, neither was entirely dependent on geriatricians—they could still provide surgery without their involvement. As well as showing that symbiotic inter‐dependencies may be asymmetrical (Liu 2011), our study highlights the possibility that such inter‐dependencies might also be ‘tight’ or ‘loose’, with the dyadic relationship between surgeon and anaesthetists remaining tight, whilst their triadic relationship with geriatricians loose. Nevertheless, the crafting of more collaborative inter‐dependencies within the healthcare eco‐system provides opportunities for geriatricians to expand and institutionalise their professional project (Pickard 2010).

We also reflect on the ontology of professional eco‐systems. Much of the literature tends to view jurisdictions like sovereign territories on a map with borders or frontiers along which competition or collaboration occurs. Our study highlights the importance of developing a more temporal‐spatial analysis of jurisdictions. In complex multi‐professional work systems, jurisdictions vary in significance at different times and places. For example, geriatricians made no claim over the core tasks of surgery or anaesthesia, especially those located in the operating room, nor ‘over’ the surgical patient; rather, they sought a more integrated role at certain times and places in the perioperative pathway, for example in assessment clinics and wards. As such, it becomes important to consider where and when jurisdictions are enacted or revoked as part of a dynamic division of labour (Allen 2002) and therefore where efforts to give up jurisdiction at certain times or places may also reflect efforts to concentrate or maintain jurisdiction at other times and places. Put simply, some territory is more valuable at certain times and places because it confers access to particular resources. This means boundary disputes can involve skirmishes between professional groups, rather than redrawing boundaries, and that ceding territory may reflect the desire to mitigate risk or discharge responsibility for unwanted duties. Future research on professional jurisdictions might consider the ‘where and when’ of jurisdiction as much as the ‘what and how’.

Finally, we discuss the practical implications of our study. With growing calls for inter‐speciality working to meet the complex care needs of an ageing population and workforce shortages (McPake et al. 2024), attention is needed to how workforce innovations will be implemented. The first is an appeal to policymakers, service managers and professional leaders to consider that workforce change will rarely occur without some degree of disharmony, and it is therefore important to devise strategies for implementing change locally. Our study suggests a combination of inter‐personal capabilities, that is, for trust building, incremental (and non‐confrontational) co‐working and the ability to evidence change, are integral to making demonstrable claims. The second is to recognise the potential impediments to quality and safety that might result from sustained inter‐speciality conflict, especially where clinical decision‐making might be partial or biased towards certain options rather than others. A growing body of research suggests that carefully planned integration of geriatrician across the perioperative pathway is associated with improved surgical outcomes, patient experience and resource utilisation (Partridge et al. 2018). More than this, there is a more fundamental question about how best to respect the individuality and autonomy of older people—supporting them in making decisions about their care by ensuring they are informed by a plurality of perspectives across multiple groups.

## 3 Conclusion

Our study examined the micro‐processes of jurisdictional change in the eco‐systems of healthcare work. It contributes to literature by showing how the expansion of professional jurisdictions involves making ‘demonstrable claims’ and engaging in ‘jurisdictional clipping’. These strategies combine discursive claims with (often hidden) material practices to make and demonstrate the value of additional expertise to incumbent professions, and also how these practices are focused on specific tasks located at the temporal and spatial peripheries of the prevailing jurisdictions. Although such strategies might result in the expansion of one jurisdiction at the expense of another, our study shows how this enabled incumbent professions to delegate marginal aspects of their work in order to concentrate on core specialist tasks. Thus, the changed systems of healthcare work might lead to new forms of collaboration whilst also reinforcing prevailing professional hierarchies.

## Author Contributions

**Justin J. Waring:** conceptualization, funding acquisition, investigation, methodology, project administration, data curation, formal analysis, writing–original draft preparation, writing–review and editing. **Graham P. Martin:** formal analysis, writing–original draft preparation, writing–review and editing.

## Ethics Statement

The research received favourable ethical review by the University of Birmingham Research Governance department.

## Conflicts of Interest

The authors declare no conflicts of interest.

## Data Availability

Anonymised data can be provided by request to the lead author.
